# Unexpected Effects of Low Doses of a Neonicotinoid Insecticide on Behavioral Responses to Sex Pheromone in a Pest Insect

**DOI:** 10.1371/journal.pone.0114411

**Published:** 2014-12-17

**Authors:** Kaouther K. Rabhi, Kali Esancy, Anouk Voisin, Lucille Crespin, Julie Le Corre, Hélène Tricoire-Leignel, Sylvia Anton, Christophe Gadenne

**Affiliations:** INRA/Université d'Angers, Neuroéthologie-RCIM, UPRES-EA 2647 USC INRA 1330, SFR 4207 QUASAV, 42, rue Georges Morel, F-49071 Beaucouzé, France; United States Department of Agriculture, Beltsville Agricultural Research Center, United States of America

## Abstract

In moths, which include many agricultural pest species, males are attracted by female-emitted sex pheromones. Although integrated pest management strategies are increasingly developed, most insect pest treatments rely on widespread use of neurotoxic chemicals, including neonicotinoid insecticides. Residual accumulation of low concentrations of these insecticides in the environment is known to be harmful to beneficial insects such as honey bees. This environmental stress probably acts as an “info-disruptor” by modifying the chemical communication system, and therefore decreases chances of reproduction in target insects that largely rely on olfactory communication. However, low doses of pollutants could on the contrary induce adaptive processes in the olfactory pathway, thus enhancing reproduction. Here we tested the effects of acute oral treatments with different low doses of the neonicotinoid clothianidin on the behavioral responses to sex pheromone in the moth *Agrotis ipsilon* using wind tunnel experiments. We show that low doses of clothianidin induce a biphasic effect on pheromone-guided behavior. Surprisingly, we found a hormetic-like effect, improving orientation behavior at the LD20 dose corresponding to 10 ng clothianidin. On the contrary, a negative effect, disturbing orientation behavior, was elicited by a treatment with a dose below the LD0 dose corresponding to 0.25 ng clothianidin. No clothianidin effect was observed on behavioral responses to plant odor. Our results indicate that risk assessment has to include unexpected effects of residues on the life history traits of pest insects, which could then lead to their adaptation to environmental stress.

## Introduction

Most animals including insects rely on olfaction to find their mating partners. In moths, which include many important agricultural pests at the larval stage, males are attracted by sex pheromones emitted by conspecific females [1]. Although integrated pest management strategies are increasingly developed [2], most insect pest treatments still rely on neurotoxic chemicals, including neonicotinoid insecticides [3]. These molecules, such as the widely used last-generation insecticide clothianidin, are known to disrupt synaptic transmission through their action on nicotinic acetylcholine receptors [4], [5]. Neonicotinoid insecticides are used extensively for the control of important agricultural crop pests through spraying, seed dressing, and soil additions [3].

The widespread use of such neurotoxic insecticides results in residual accumulation of low concentrations in the environment [6]. This environmental stress probably acts as an “info-disruptor” by modifying the chemical communication system [7], and therefore decreases chances of reproduction in target insects. Sublethal doses of insecticides have been shown to alter various behaviors of beneficial insects such as honey bees [8]. Previous data likewise demonstrate that low doses of certain insecticides disrupt the behavioral response of pest insects to sex pheromone in a few species [9].

However, very low doses of pollutants can, on the contrary, induce an unexpected increase of reproduction abilities in target insects, which could allow them to bypass this stress, favoring the development of insecticide resistance or adaptation [10]. The phenomenon of hormesis, defined as a biphasic response following exposure to a given toxicant with beneficial effects at low-dose exposure and adverse effects at high-dose exposure [11], might be one explanation for these observations. Examples of the toxicological phenomenon of hormesis have been reported for many types of biological and pathological processes including microbial responses, plant responses, reproductive traits, and various stages of cancer [12], [13]. In insects, insecticide-induced hormesis in developmental and reproductive life traits (such as growth stimulation (weight), enhanced pupation, decrease of pupal mortality, increased fecundity and longevity, and increase of oviposition) has likewise been observed following treatments with classical non-neonicotinoid substances such as carbamates and organophosphates [14], [15]. Very little is known on the possible hormetic effect of insecticides in general on olfactory-guided behavior ([14] and references therein) and no study has dealt so far with the effects of neonicotinoid treatments on olfaction.

The black cutworm, *Agrotis ipsilon* (Hufnagel) (Lepidoptera: Noctuidae), is a worldwide pest of many crops including corn and may cause severe stand losses and injury to corn seedlings [16]. In a few moth species including *A. ipsilon*, evidence is accumulating that behavioral and neural responses to odors can be modulated by age, physiological state or previous experience [17]–[20]. The well-described olfactory plasticity in *A. ipsilon* makes this species an excellent model to study effects of low insecticide doses on olfactory-guided behavior.

Here we tested the effect of low doses of clothianidin (0.1 to 20 ng per moth) on the behavioral responses to sex pheromone in *A. ipsilon* males in wind tunnel experiments. This neonicotinoid has insecticidal effects on a broad range of insect pests including plant-sucking insects [3], [21] and is widely used against *A. ipsilon* in foliar, soil and seed treatments [3], [22], [23]. Contrary to previous studies using only 1–3 doses of insecticides [24], [25], we evaluated dose-dependent effects of clothianidin on *A. ipsilon* behavior in more detail, by testing 8 doses ranging from the LD30 down to below the LD0. We show that acute oral treatments with low doses of clothianidin induced a biphasic effect on pheromone-guided behavior. A positive effect improving orientation behavior was found at a dose of 10 ng clothianidin per moth, corresponding to the LD20, whereas a negative effect, disturbing orientation behavior, was elicited by a treatment with 0.25 ng per moth, corresponding to a dose 10 times lower than the LD0. The effects of both clothianidin doses were restricted to pheromone responses and did not modify responses to plant odor.

## Results

### Acute toxicity assay and sublethal dose determination

**Figure 1:**
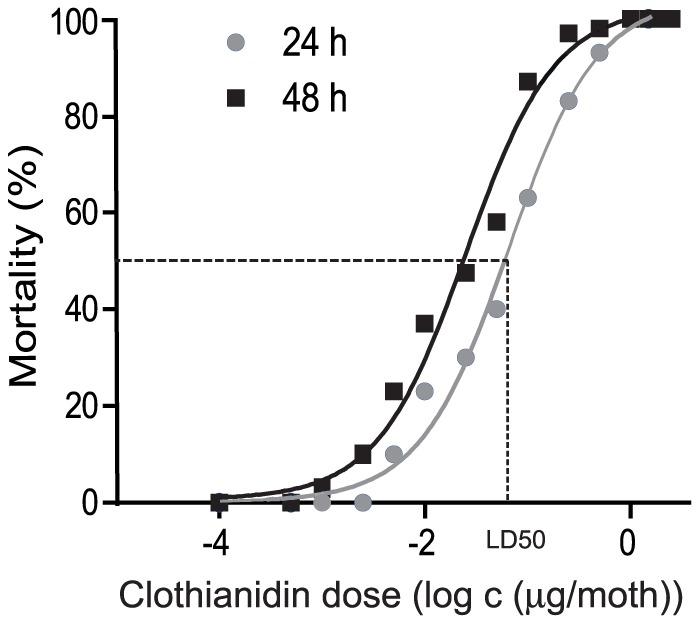
Toxicity of acute oral clothianidin treatment for adult *A. ipsilon* males. Four-day-old males were intoxicated with a range of concentrations of clothianidin (0.1 ng–2.5 µg/moth). The number of surviving males was recorded 24 h and 48 h after intoxication. The median lethal dose (LD50) was determined at 69±0.04 ng and 29±0.07 ng/moth 24 h and 48 h after treatment respectively. N = 50 for each dose.

Four day-old sexually mature males were fed individually with clothianidin or control solutions (solvent dimethyl sulfoxide [DMSO] or sucrose) and mortality was recorded 24 h and 48 h later. For most males, the solution containing clothianidin was quickly ingested (a few seconds). No mortality was observed in control groups (n = 50 for sucrose and solvent groups). The theoretical lethal dose 50 (LD50: dose resulting in 50% mortality post-treatment) was determined by dose-response assays ranging from 0.1 ng to 2.5 µg/moth (n = 50 for each dose) (Fig. 1) and was found to be 69±0.04 ng/moth and 29±0.07 ng/moth 24 h and 48 h after treatment, respectively. Before dying, intoxicated insects exhibited trembling and incapacity to move.

Eight low doses that resulted in 30% of mortality or less 24 h after treatment were used for behavioral tests. The LD30, LD25, LD20, and LD10 were determined to be 25, 15, 10 and 5 ng/moth respectively. The LD0 was determined as 2.5 ng/moth, and we also included treatments with 3 doses below the LD0: 1, 0.25, and 0.1 ng/moth.

### Effect of DMSO on flight ability and pheromone responses

The effect of solvent treatments on male behavior was determined. More than 50 males were tested for each dose of DMSO or sucrose control. No statistical difference in the flight ability, general flight activity and pheromone responses (i.e. oriented flight towards the pheromone) was observed after treatment with any of the 8 DMSO doses used for the preparation of clothianidin solutions as compared with control sucrose-treated males (G = 18.8, df = 15, P = 0.22; G = 11.16, df = 15, P = 0.74 and G = 18.17, df = 15, P = 0.25 for flight ability, general flight activity and pheromone responses respectively) (File S1).

In the following experiments, the flight ability and pheromone responses of males intoxicated with clothianidin were thus compared to those of males treated with the corresponding DMSO dose.

### Effect of clothianidin on flight ability

More than 50 males were tested if they were able to fly for each dose of clothianidin or DMSO. No significant differences in the flight ability between clothianidin- and DMSO-treated males were observed after intoxication with doses of 2.5 ng/moth and lower (2.5 ng/moth: G = 0.58, df = 1, P = 0.44; 1 ng/moth: G = 0.06, df = 1, P = 0.79; 0.25 ng/moth: G = 0.05, df = 1,P = 0.81; 0.1 ng/moth: G = 0.8, df = 1, P = 0.36) (Figure A in S2 File). On the contrary, exposure to doses of 5 ng clothianidin and above decreased the proportion of males able to fly (25 ng/moth: G = 8.7, df = 1, P = 0.003; 15 ng/moth: G = 25.03, df = 1, P = 5.62.10^−7^; 10 ng/moth: G = 4.01, df = 1, P = 0.04; 5 ng/moth: G = 6.55, df = 1, P = 0.01) (Figure A in S2 File). Only males that were able to fly were considered for the behavioral analyses described below.

### Effect of clothianidin on pheromone responses

**Figure 2:**
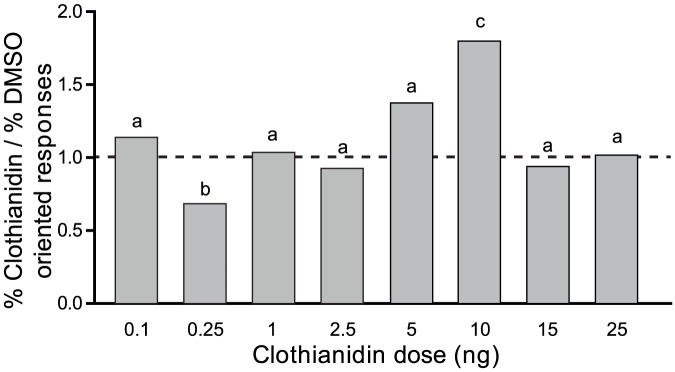
Effect of low doses of clothianidin on oriented responses of *A. ipsilon* males towards a 20-ng sex pheromone blend. The percentage of responses of clothianidin-treated males divided by that of DMSO-treated males is represented. The 0.25-ng dose induced a significant decrease in oriented responses whereas the 10-ng dose induced a significant increase in sex pheromone oriented responses. Bars with same letters are not significantly different (G-test, P<0.05). The dashed line represents an equal response of males treated with clothianidine and the corresponding DMSO dose. N>50 for all groups.

As there was no significant difference between the oriented responses of control males and males treated with DMSO (see above, File S1), we show the effect of clothianidin on the male oriented response as compared to the corresponding DMSO responses (i.e. the percentage of responses in clothianidin-treated males divided by the percentage of responses of males treated with the corresponding DMSO doses) (Fig. 2). Acute treatment with 2.5 ng, 1 ng, or 0.1 ng clothianidin (<LD0) did not affect the oriented behavioral response to 20 ng of the pheromone (2.5 ng: G = 0.17, df = 1, P = 0.67; 1 ng: G = 0.05, df = 1, P = 0.81; 0.1 ng: G = 0.51, df = 1, P = 0.47) (Fig. 2). However, significantly fewer males responded to the pheromone after treatment with 0.25 ng/moth as compared to DMSO-treated controls (G = 4.34, P = 0.04) (Fig. 2).

In contrast, significantly more males responded to the pheromone after treatment with 10 ng/moth (LD20) as compared to DMSO-treated males (G = 12.19; df = 1; P = 0.0005) (Fig. 2). To check for typical characteristics of a hormetic effect, we also treated male moths with doses close to the LD20 (25, 15 and 5 ng/moth corresponding to LD30, LD25 and LD10). For these doses we observed no significant difference in behavioral pheromone responses compared to DMSO-treated males (25 ng/moth: G = 0.01, df = 1, P = 0.9; 15 ng/moth: G = 0.18, df = 1, P = 0.66; 5 ng/moth: G = 2.87, df = 1, P = 0.09) (Fig. 2). For all tested groups, the general flight activity was high and not statistically different between treated and control males (G test: G = 11.14, df = 15, P = 0.74) and between groups treated with different doses of clothianidin (G = 2.72, df = 7, P = 0.90) (Figure B in S2 File).

**Figure 3:**
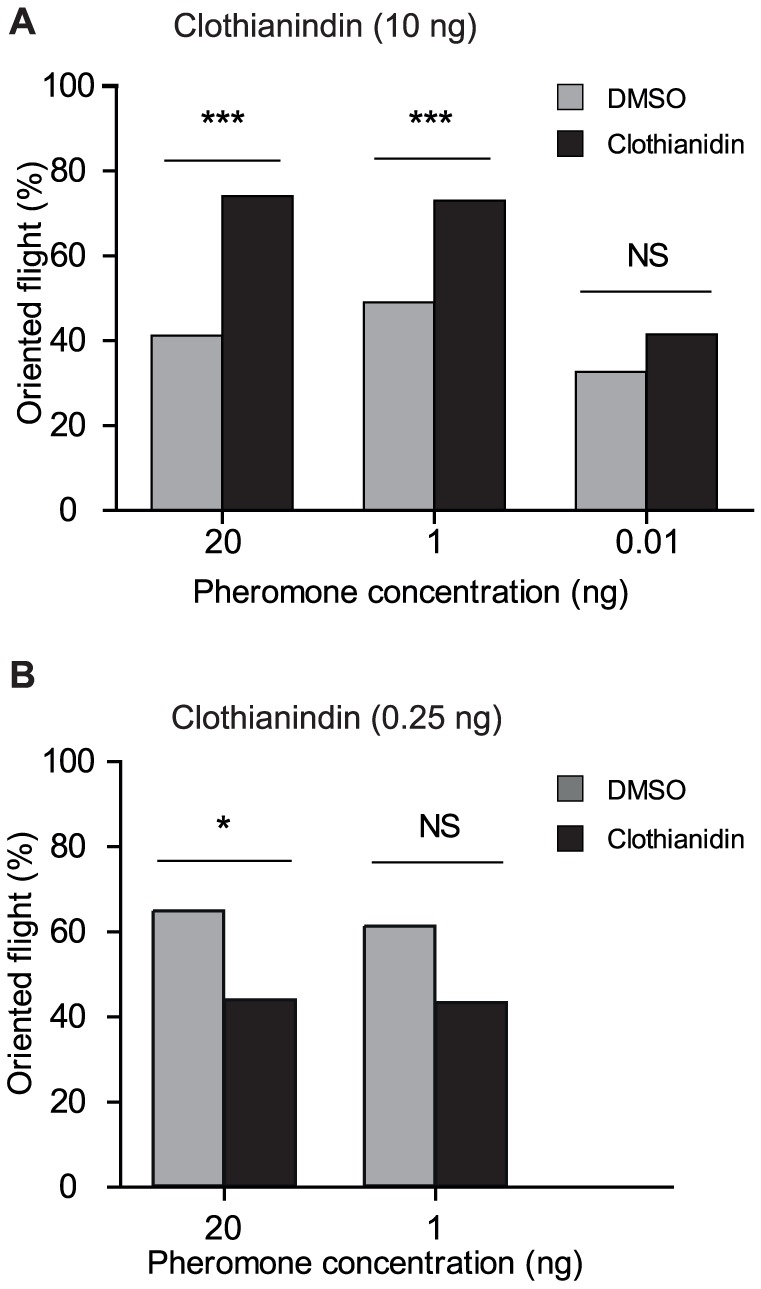
Effect of the 10 ng (A) and 0.25 ng (B) doses of clothianidin on responses of *A. ipsilon* males to different doses of the sex pheromone blend. The effect of the 10 ng clothianidin treatment was confirmed with a 1 ng dose of pheromone but not with a 0.01 ng dose. The effect of the 0.25 ng clothianidin dose was not significant when using a 1 ng pheromone stimulus. N>50 for all groups, G-test, * P<0.05; ** P<0.01; *** P<0.001.

In order to confirm the observed behavioral increase in pheromone response of males after the 10-ng clothianidin treatment (LD20), we examined behavioral responses of treated and untreated males to lower doses of pheromone (Fig. 3A). The effect observed with the 20 ng pheromone dose was confirmed with the 1 ng pheromone dose (G = 6.25, df = 1, P = 0.01) but no significant effect was observed with the 0.01 ng pheromone dose (G = 0.86, df = 1, P = 0.35) (Fig. 3A). Similarly, in order to verify the decreased response levels observed after the 0.25-ng clothianidin treatment, we also tested the pheromone response of males treated with this dose of clothianidin to a lower dose of pheromone. With a 1 ng pheromone stimulus we did not observe a significant decrease in the proportion of responding males as compared to DMSO-treated males (G = 3.36, df = 1, P = 0.066) (Fig. 3B).

### Effects of clothianidin on plant odor responses

**Figure 4:**
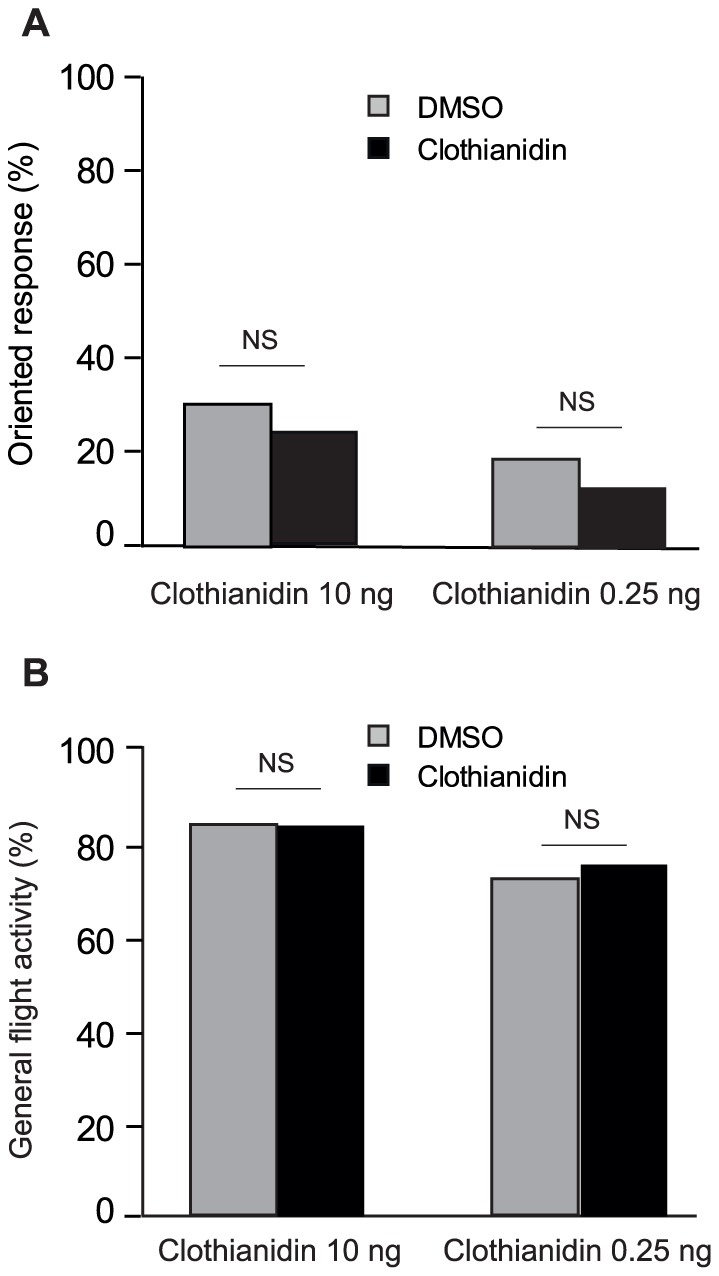
Effect of the 10-ng and 0.25-ng low doses of clothianidin on oriented responses (A) and general flight activity (B) of *A. ipsilon* males in presence of a linden flower extract. Responses to the linden flower extract were not significantly different between clothianidin- and DMSO-treated males for both 0.25 ng and 10 ng clothianidin. N>50 for all groups, G-test, P<0.05.

We also tested the effect of low doses of clothianidin on the responses of at least 50 intoxicated males/dose to a linden flower extract (see Methods for details). For this, we selected the two clothianidin doses which induced the observed positive (10 ng) and negative (0.25 ng) effects on sex pheromone responses. The general flight activity and the oriented responses towards the linden flower extract were not statistically different between intoxicated and control males (10 ng/moth: G = 0.56, df = 1, P = 0.46; 0.25 ng/moth: G = 0.69, df = 1, P = 0.4) (Fig. 4).

## Discussion

In the present study we show that a low dose (LD20) of a neonicotinoid insecticide can increase behavioral responses to sex pheromone in the surviving individuals of a pest insect and a very low dose, while not causing any mortality, can decrease sex pheromone responses. Whereas the latter effect probably reinforces the efficiency of insecticide treatments, the improved pheromone responses observed with the LD20 dose could threaten the efficiency of these treatments. However, this dose, although increasing the pheromone response, reduced the survival rate and flight capacity of the intoxicated moths. Therefore the potential overall gain in the reproductive capacity of males treated with this dose might be minimal. Our results indicate that an insect could modulate its olfactory system to bypass environmental anthropogenic changes, such as the growing presence of insecticide residues.

During acute oral intoxication treatments, *A. ipsilon* males readily and quickly ingested the provided clothianidin solutions, confirming that this neonicotinoid is not a feeding deterrant [23]. Symptoms of intoxication were similar to those described for most neurotoxic insecticides [8], [26]. The *A. ipsilon* LD50 24 h after acute oral intoxication (69 ng/moth) with clothianidin was in the same order of magnitude as that of the honey bee (3–50 ng/bee) [27]–[29].

Previous studies have shown that sublethal doses of neonicotinoids induce various effects on development and life traits, including behavior, in many insects. These effects are mainly negative, and have been observed principally in beneficial insects such as honey bees (for example [8], [30], but also in ants [31], aphids [24], [32], [33], moths [23], [34], [35], bugs [25], [36], [37], and flies [38]. However, effects of neonicotinoids on responses to behaviorally relevant odors have not been investigated in pest insects so far. Here we show that intoxication with very low sublethal doses of clothianidin (1/10 of the LD0 dose: 0.25 ng/moth) decreased the percentage of *A. ipsilon* males responding to 20 ng of the sex pheromone, corresponding to the emission of 20 females [39], even though this 0.25 ng-clothianidin dose did not affect locomotor behavior, and males were able to fly as well as control males. A few studies have already shown “negative” effects of other types of insecticides on sexual behavior of pest insects, i.e. the behavioral response to sex pheromone [9]. For example, in the oriental fruit moth, *Grapholita molesta,* the carbamate carbaryl disrupted zigzagging upwind flight, and the organochloride chlordimeform interfered with all sequences of flight and courtship display [40]. In the corn borer *Ostrinia furnacalis*, treatments with the organophosphate malathion and the pyrethroid deltamethrin decreased the ability of males to respond to the female-produced sex pheromone [41], [42].

Surprisingly, clothianidin treatments at the LD20 dose, a higher dose than that which elicited the observed negative effects, increased the percentage of *A. ipsilon* males responding to the sex pheromone. This effect on the olfactory system can be compared with effects of the cyano-substituted neonicotinoid acetamiprid at doses below LD0, which elicits increased sensitivity to antennal stimulation with sucrose similarly to nicotine injections in the honey bee [43], [44]. Very low doses of other insecticides such as chlordimeform have also shifted male moth behavioral sensitivity to low doses of pheromone in *Trichoplusia ni* and *G. molesta* [40], [45]. Another case of increased behavioral responses to olfactory signals after acute insecticide treatments was found in parasitoid wasps. Here sublethal treatments at an LD 20 dose of the organophosphorous and pyrethroid compounds chlorpyrifos and deltamethrin elicited for example improved responses to host kairomones [46], [47]. Unexpected positive effects of sublethal doses of the neonicotinoid imidacloprid on various biological traits such as fecundity and longevity have also been observed in aphids and hemipterans [25], [32], [48].

Such described positive effects of low doses of a toxicant characterize the hormesis effect. Classically, a hormetic response is characterized by a high dose inhibition and a low dose stimulation of a given compound, represented by an inverted U-shape dose-response [10], [11], [49]. Evidence of such a biphasic dose–response relationship for pesticides has been found in many insect species [14]. It is recognized as one of the potential causes underlying pest resurgence and secondary pest outbreaks [15], [50]–[52]. In the present study, low doses of clothianidin induced a biphasic effect on the behavioral response to sex pheromone in the moth *A. ipsilon*: negative and positive effects on pheromone responses were obtained with very low (<LD0) and higher (LD20) doses respectively, whereas even higher doses did not affect behavior. To our knowledge, this is the first report of such low dose inhibition and high dose stimulation of a single biological trait following intoxication by a pollutant.

The robustness of our behavioral assay, coupled with the fact that significant positive effects were still found following tests with lower doses of pheromone (1 ng), clearly indicates that the 10 ng clothianidin dose provides the “low-dose stimulation” defined by the hormesis phenomenon. However, declaring that we have a concrete example of hormesis based upon this evidence would be premature for two reasons. Firstly we observed a negative effect at an even lower dose of clothianidin treatment. Secondly, although there was a tendency for a positive behavioral effect following treatment with the 5-ng dose of clothianidin, we did not find a true significant positive effect with a neighboring toxicant dose (5 or 15 ng), a necessary criterion that characterizes hormesis [13]. Our results can thus be interpreted as a hormetic-like effect. We are currently investigating the neurobiological bases of these behavioral effects, using electrophysiological and molecular approaches. In particular, we are testing possible changes in spontaneous activity and in the sensitivity of AL neurons to pheromone under specific clothianidin treatments using intracellular recordings of AL neurons. Additionally, we are investigating the expression levels of different receptor subunits within the *A. ipsilon* brain following treatment with the positive (10 ng) and negative (0.25 ng) sublethal doses using qPCR analysis.

From a mechanistic point of view, the biphasic effect of sublethal doses on male pheromone-guided behavior could be explained by differential affinity of clothianidin to nicotinic receptor subtypes, constituted of different subunits within the olfactory system [53]. Several facts reported in the literature support this hypothesis. In other insects, such as the brown planthopper, the subunit composition of nicotinic receptors has been shown to influence the affinity to the neonicotinoid imidacloprid: while the β1 subunit is required for imidacloprid binding, it is the identity of the remaining subunits associated with β1 that determines binding site affinity [54]. In hybrid rat/Drosophila nicotinic receptors, imidacloprid was also found to show differential binding affinities according to the Drosophila subunit co-expressed with the rat subunit in a Drosophila cell line [55]. For clothianidin, different binding sites of nAChRs have recently been identified, which would allow for different affinities within the same insect [56]. Indeed certain nicotinic receptor subunits have been shown to be differentially expressed in different parts of the honey bee brain, including areas treating olfactory information such as the antennal lobes [57], [58], and could thus explain different affinities to insecticides in different parts of the nervous system. In our case, different neuron types within the moth AL might express different nACh receptor types, consisting of different subunit arrangements leading to different affinity for clothianidin. These differentially affected neuron types within the AL network might exhibit dissimilar activity following treatments with different doses of clothianidin. Different sets of nicotinic receptor subunits between the MGC and “ordinary” glomeruli of the AL might also explain why behavioral responses to plant odors are not affected by the tested clothianidin treatments.

## Methods

### Insects

Adult males of *A. ipsilon* Hufnagel originated from a laboratory colony established in Bordeaux and transferred to Angers. Wild insects are introduced into the colony each spring to maintain genetic diversity and overall health of the colony. Insects were reared on an artificial diet [59] in individual cups until pupation. Pupae were sexed, and males and females were kept separately at 22°C in an inversed light-dark cyle (16 h: 8 h light: dark photoperiod) with the scotophase starting at 10 am. Newly emerged adults were removed every day from hatching containers and were given access to a 20% sucrose solution *ad libitum*. The day of emergence was considered as Day 0. Four- and five-day old virgin males of *A.ipsilon*, were used in this study.

### Chemicals

Clothianidin (>99% purity, Sigma-Aldrich, Saint-Quentin Fallavier, France) was first dissolved in dimethyl sulfoxide (DMSO) and dilutions were then prepared with a 20% sucrose solution. The DMSO concentration in the most concentrated clothianidin solution used was at 2%. Fresh solutions were prepared weekly as needed from frozen aliquots of the stock solution.

For wind tunnel experiments, we used an artificial pheromone blend containing (Z)-7-dodecenyl acetate (Z7–12:Ac), (Z)-9-tetradecenyl acetate (Z9–14:Ac), and (Z)-11-hexadecenyl acetate (Z11–16:Ac) (Sigma–Aldrich) at a ratio of 4∶1∶4 as an attractant [39], [60], or a linden flower extract (*Tilia tormentosa*, 55% purity, Boiron laboratories, Sainte-Foy les Lyon, France) [19].

### Clothianidin intoxication

In order to control the insecticide dose received by each individual moth, oral acute exposure was chosen as the method for delivery. Exposure to clothianidin was accomplished by feeding 4-days old males with the various doses of clothianidin. We chose 4-days old males for treatments, because the effects of low doses of clothianidin were assessed 24 h after treatment (day-5), which corresponds to the optimal age for behavioral responses to sex pheromone in wind tunnel experiments [61]. Males were transferred from their rearing chamber before the onset of the scotophase under a ventilated fume hood. They were then placed in plastic pipette tips, with just the head protruding, to limit their movements. Using forceps, the proboscis was extended to a 10 µl drop of the concentration to be tested and held there until the moth had finished ingesting the entire drop. Control solutions of DMSO corresponding to the concentration of DMSO in each clothianidin treatment were delivered in an identical manner.

To test the impact of this delivery method, initial experiments were performed comparing the behavioral responses of untreated moths, which were fed with 10 µl of sucrose solution under the same conditions of restraint. No mortality was observed in sucrose-treated males, and no significant difference in behavioral responses were found between untreated control moths (n = 243) and sucrose-fed moths (n = 231) (G = 0.91; df = 1; P = 0.33).

Once the males had ingested the presented solution, they were placed in individual plastic cups and transferred to a climate chamber different from the rearing room for toxicological (24 h and 48 h later) and behavioral tests (24 h later). Intoxication of a batch of 30 moths took approximately 30 min.

### Mortality tests

Clothianidin treatments were done with 15 different concentrations of clothianidin (0.1 ng–2.5 µg/moth) and the corresponding DMSO solutions. Each day of intoxication, clothianidin as well as DMSO and sucrose solutions were applied to a group of 10 moths each. Evaluation of mortality was based on the number of males exhibiting no movement even when prodded or touched, 24 h and 48 h after treatment. A total of 50 males for each group were treated for each concentration.

### Wind tunnel experiments

Behavioral tests were performed using a 2 m-long flight tunnel during the middle of the scotophase (4–7 h after lights off) under red light illumination as described previously [19]. Four-day-old males were treated with 8 different doses of clothianidin (0.1; 0.25; 1; 2.5; 5; 10; 15; 20 ng) or the corresponding DMSO dose. A third group of control males remained untreated. The behavioral responses of 5-day-old intoxicated or control males were tested. Environmental conditions during the bioassay were held constant: 22°C, 50±10% relative humidity, and a wind speed of 0.3 m^s−1^. All experiments were performed double-blindly to avoid biased observations, i. e. the observer did not know the treatment of the male. A cage containing a single experimental male was introduced in the wind tunnel. After 30 s, during which the male adjusted to the airflow, a filter paper containing the odor stimulus was placed 160 cm upwind from the cage. Pheromone stimulation was performed with an artificial pheromone blend as described above. Different concentrations of this pheromone blend (20, 1, and 0.01 ng in 10 µl) were used for behavioral tests. Fifty µl of the undiluted linden flower extract were used to test behavioral responses to the plant odor.

Moth behavior was observed during 3 min. Partial flight (characteristic zigzag flight for half the length of the wind tunnel), complete flight (characteristic flight throughout the wind tunnel, arriving within 2 cm of the odor source), and landing on the odor source were scored as oriented responses. Behaviors such as random flight, walking, and no movement were scored as “no oriented response”. Oriented and random flights were pooled to assess general flight activity. Flight ability was tested by gently tossing the males that had not flown during their trials in the air. At the end of the experiments, all tested males were discarded in dedicated toxicology containers. For each day of experiments, we tested individuals of the three groups of males (clothianidin-, DMSO-, and sucrose-treated males).

### Statistical analysis

For behavioral assays, statistical differences (p≤0.05) were evaluated among groups (control sucrose group versus control DMSO group, and DMSO group versus clothianidin-intoxicated group) using a R x C test of independence by means of a G-test and applying the William's correction [62].

## Supporting Information

S1 File **Effect of acute oral DMSO treatment on oriented flight (A), flight ability (B), and general flight activity (C) of adult** ***A. ipsilon*** **males.** Altogether there was no statistical difference between DMSO- and sucrose-treated males for flight ability, general flight activity and oriented pheromone response (G-test, P>0.05).
(EPS)

S2 File **Effects of low doses of clothianidin on the flight ability (A) and general flight activity (B) of** ***A. ipsilon*** **males.** **A** The doses of 25, 15, 10 and 5 ng/moth negatively affected the locomotor behavior of males. **B** No difference in general flight activity was observed after clothianidin treatment. N>50 for all groups, G-test, P≤0.05.
(EPS)

## References

[1] Roelofs WL (1995) Chemistry of sex attraction. Proc Natl Acad Sci USA 92:44–49. 7816846 10.1073/pnas.92.1.44 PMC42814

[2] Suckling DM, Stringer LD, Stephens AEA, Woods B, Williams DG, et al (2014) From integrated pest management to integrated pest eradication: technologies and future needs. Pest Manage Sci 70:179–189. 10.1002/ps.3670 24155254

[3] Jeschke P, Nauen R, Schindler M, Elbert A (2011) Overview of the Status and Global Strategy for Neonicotinoids. J Agric Food Chem 59:2897–2908. 20565065 10.1021/jf101303g

[4] Casida JE, Durkin KA (2013) Neuroactive insecticides: targets, selectivity, resistance, and secondary effects. Annu Rev Entomol 58:99–117. 23317040 10.1146/annurev-ento-120811-153645

[5] Matsuda K, Buckingham S, Kleier D, Rauh J, Grauso M, et al (2001) Neonicotinoids: insecticides acting on insect nicotinic acetylcholine receptors. Trends Pharmacol Sci 22:573–580. 11698101 10.1016/s0165-6147(00)01820-4

[6] Goulson D (2013) REVIEW: An overview of the environmental risks posed by neonicotinoid insecticides. J Appl Ecol 50:977–987.

[7] Lurling M, Scheffer M (2007) Info-disruption: pollution and the transfer of chemical information between organisms. Trends Ecol Evol 22:374–379. 17433848 10.1016/j.tree.2007.04.002

[8] Desneux N, Decourtye A, Delpuech J-M (2007) The sublethal effects of pesticides on beneficial arthropods. Annu Rev Entomol 52:81–106. 16842032 10.1146/annurev.ento.52.110405.091440

[9] Haynes KF (1988) Sublethal effects of neurotoxic insecticides on insect behavior. Annu Rev Entomol 33:149–168. 3277528 10.1146/annurev.en.33.010188.001053

[10] Guedes NMP, Tolledo J, Correa AS, Guedes RNC (2010) Insecticide-induced hormesis in an insecticide-resistant strain of the maize weevil, *Sitophilus zeamais* . J Appl Entomol 134:142–148.

[11] Calabrese EJ, Baldwin LA (2002) Defining Hormesis. Hum Exp Toxicol 21:91–97. 12102503 10.1191/0960327102ht217oa

[12] Calabrese EJ (2009) The road to linearity: why linearity at low doses became the basis for carcinogen risk assessment. Arch Toxicol 83:203–225. 19247635 10.1007/s00204-009-0412-4

[13] Calabrese EJ, Baldwin LA (2001) The frequency of U-shaped dose responses in the toxicological literature. Toxicol Sci 62:330–338. 11452146 10.1093/toxsci/62.2.330

[14] Cutler GC (2013) Insects, insecticides and hormesis: evidence and considerations for study. Dose-Response 11:154–177. 23930099 10.2203/dose-response.12-008.Cutler PMC3682195

[15] Guedes RNC, Cutler GC (2013) Insecticide-induced hormesis and arthropod pest management. Pest Manage Sci 70:690–697. 10.1002/ps.3669 24155227

[16] Rings RW, Arnold FJ, Keaster AJ, Musick GJ (1974) Worldwide annotated bibliography of the black cutworm *Agrotis ipsilon*, Hufnagel. Ohio Agric Res Dev Cent Res Circ: 1–106.

[17] Anton S, Dufour MC, Gadenne C (2007) Plasticity of olfactory-guided behaviour and its neurobiological basis: lessons from moths and locusts. Entomol Exp Appl 123:1–11.

[18] Anton S, Evengaard K, Barrozo RB, Anderson P, Skals N (2011) Brief predator sound exposure elicits behavioral and neuronal long-term sensitization in the olfactory system of an insect. Proc Natl Acad Sci USA 108:3401–3405. 21300865 10.1073/pnas.1008840108 PMC3044404

[19] Barrozo RB, Gadenne C, Anton S (2010) Switching attraction to inhibition: mating-induced reversed role of sex pheromone in an insect. J Exp Biol 213:2933–2939. 20709921 10.1242/jeb.043430

[20] Saveer AM, Kromann SH, Birgersson G, Bengtsson M, Lindblom T, et al (2012) Floral to green: mating switches moth olfactory coding and preference. P Roy Soc B 279:2314–2322. 10.1098/rspb.2011.2710 PMC3350682 22319127

[21] Nauen R, Ebbinghaus-Kintscher U, Salgado VL, Kaussmann M (2003) Thiamethoxam is a neonicotinoid precursor converted to clothianidin in insects and plants. Pestic Biochem Physiol 76:55–69.

[22] Elbert A, Haas M, Springer B, Thielert W, Nauen R (2008) Applied aspects of neonicotinoid uses in crop protection. Pest Manage Sci 64:1099–1105. 10.1002/ps.1616 18561166

[23] Kullik SA, Sears MK, Schaafsma AW (2011) Sublethal Effects of Cry 1F Bt Corn and Clothianidin on Black Cutworm (Lepidoptera: Noctuidae) Larval Development. J Econ Entomol 104:484–493. 21510196 10.1603/ec10360

[24] Shi X, Jiang L, Wang H, Qiao K, Wang D, et al (2011) Toxicities and sublethal effects of seven neonicotinoid insecticides on survival, growth and reproduction of imidacloprid-resistant cotton aphid, *Aphis gossypii* . Pest Manage Sci 67:1528–1533. 10.1002/ps.2207 21714058

[25] Tan Y, Biondi A, Desneux N, Gao X-W (2012) Assessment of physiological sublethal effects of imidacloprid on the mirid bug *Apolygus lucorum* (Meyer-Dür). Ecotoxicology 21:1989–1997. 22740097 10.1007/s10646-012-0933-0

[26] Aliouane Y, El Hassani AK, Gary V, Armengaud C, Lambin M, et al (2009) Subchronic exposure of honeybees to sublethal doses of pesticides: effects on behavior. Environ Toxicol Chem 28:113–122. 18700810 10.1897/08-110.1

[27] Decourtye A, Devillers J (2010) Ecotoxicity of Neonicotinoid Insecticides to Bees. Insect Nicotinic Acetylcholine Receptors 683:85–95. 10.1007/978-1-4419-6445-8_8 20737791

[28] Laurino D, Manino A, Patetta A, Porporato M (2013) Toxicity of neonicotinoid insecticides on different honey bee genotypes. Bull Insectol 66:119–126.

[29] Laurino D, Porporato M, Patetta A, Manino A (2011) Toxicity of neonicotinoid insecticides to honey bees: laboratory tests. Bull Insectol 64:107–113.

[30] Henry M, Beguin M, Requier F, Rollin O, Odoux J-F, et al (2012) A Common Pesticide Decreases Foraging Success and Survival in Honey Bees. Science 336:348–350. 22461498 10.1126/science.1215039

[31] Barbieri RF, Lester PJ, Miller AS, Ryan KG (2013) A neurotoxic pesticide changes the outcome of aggressive interactions between native and invasive ants. P Roy Soc B 280:20132157. 10.1098/rspb.2013.2157 PMC3813335 24266038

[32] Cutler GC, Ramanaidu K, Astatkie T, Isman MB (2009) Green peach aphid, *Myzus persicae* (Hemiptera: Aphididae), reproduction during exposure to sublethal concentrations of imidacloprid and azadirachtin. Pest Manage Sci 65:205–209. 10.1002/ps.1669 19089851

[33] Miao J, Du Z-B, Wu Y-Q, Gong Z-J, Jiang Y-L, et al (2014) Sub-lethal effects of four neonicotinoid seed treatments on the demography and feeding behaviour of the wheat aphid *Sitobion avenae* . Pest Manage Sci 70:55–59. 10.1002/ps.3523 23457039

[34] Ahmad S, Ansari MS, Ahmad N (2013) Acute toxicity and sublethal effects of the neonicotinoid imidacloprid on the fitness of *Helicoverpa armigera* (Lepidoptera: Noctuidae). Int J Trop Insect Sci 33:264–275.

[35] Saber M, Parsaeyan E, Vojoudi S, Bagheri M, Mehrvar A, et al (2013) Acute toxicity and sublethal effects of methoxyfenozide and thiodicarb on survival, development and reproduction of *Helicoverpa armigera* (Lepidoptera: Noctuidae). Crop Protect 43:14–17.

[36] Bao H, Liu S, Gu J, Wang X, Liang X, et al (2009) Sublethal effects of four insecticides on the reproduction and wing formation of brown planthopper, Nilaparvata lugens. Pest Manage Sci 65:170–174. 10.1002/ps.1664 18937216

[37] Sohrabi F, Shishehbor P, Saber M, Mosaddegh MS (2011) Lethal and sublethal effects of buprofezin and imidacloprid on *Bemisia tabaci* (Hemiptera: Aleyrodidae). Crop Prot 30:1190–1195.

[38] Hu XP, Prokopy RJ (1998) Lethal and sublethal effects of imidacloprid on apple maggot fly, *Rhagoletis pomonella* Walsh (Dipt., Tephritidae). J Appl Entomol 122:37–42.

[39] Picimbon JF, Gadenne C, Bécard JM, Clément JL, Sreng L (1997) Sex pheromone of the french black cutworm moth, *Agrotis ipsilon* (Lepidoptera:Noctuidae): identification and regulation of a multicomponent blend. J Chem Ecol 23:211–230.

[40] Linn J, C E, Roelofs WL (1984) Sublethal effects of neuroactive compounds on pheromone response thresholds in male oriental fruit moths. Arch Insect Biochem Physiol 1:331–344.

[41] Wei H, Du J (2004) Sublethal effects of larval treatment with deltamethrin on moth sex pheromone communication system of the Asian corn borer, *Ostrinia furnacalis* . Pestic Biochem Physiol 80:12–20.

[42] Zhou H, Du J, Huang Y (2005) Effects of sublethal doses of malathion on responses to sex pheromones by male Asian corn borer moths, *Ostrinia furnacalis* (Guenée). J Chem Ecol 31:1645–1656. 16222799 10.1007/s10886-005-5804-1

[43] El Hassani AK, Dacher M, Gary V, Lambin M, Gauthier M, et al (2008) Effects of sublethal doses of acetamiprid and thiamethoxam on the behavior of the honeybee (*Apis mellifera*). Arch Environ Contam Toxicol 54:653–661. 18026773 10.1007/s00244-007-9071-8

[44] Thany SH, Gauthier M (2005) Nicotine injected into the antennal lobes induces a rapid modulation of sucrose threshold and improves short-term memory in the honeybee *Apis mellifera* . Brain Res 1039:216–219. 15781066 10.1016/j.brainres.2005.01.056

[45] Linn CE, Roelofs WL (1985) Multiple effects of octopamine and chlordimeform on pheromone response thresholds in the cabbage looper moth, *Trichoplusia ni* . Pestic Sci 16:445–446.

[46] Delpuech J, Bardon C, Bouletreau M (2005) Increase of the behavioral response to kairomones by the parasitoid wasp *Leptopilina heterotoma* surviving insecticides. Arch Environ Contam Toxicol 49:186–191. 16082580 10.1007/s00244-004-0158-1

[47] Komeza N, Fouillet P, Bouletreau M, Delpuech J (2001) Modification, by the insecticide chlorpyrifos, of the behavioral response to kairomones of a parasitoid wasp, *Leptopilina boulardi* . Arch Environ Contam Toxicol 41:436–442. 11598780 10.1007/s002440010269

[48] Yu Y, Shen G, Zhu H, Lu Y (2010) Imidacloprid-induced hormesis on the fecundity and juvenile hormone levels of the green peach aphid *Myzus persicae* (Sulzer). Pestic Biochem Physiol 98:238–242.

[49] Calabrese EJ, Baldwin LA (2003) Hormesis: the dose-response revolution. Annu Rev Pharmacol Toxicol 43:175–197. 12195028 10.1146/annurev.pharmtox.43.100901.140223

[50] Cohen E (2006) Pesticide-mediated homeostatic modulation in arthropods. Pestic Biochem Physiol 85:21–27.

[51] Hardin M, Benrey B, Coll M, Lamp W, Roderick G, et al (1995) Arthropod pest resistance - an overview of potential mechanisms Crop Protect. 14:3–18.

[52] Morse J (1998) Agricultural implications of pesticide-induced hormesis of insects and mites. Hum Exp Toxicol 17:266–269. 9663935 10.1177/096032719801700510

[53] Millar NS, Gotti C (2009) Diversity of vertebrate nicotinic acetylcholine receptors. Neuropharmacology 56:237–246. 18723036 10.1016/j.neuropharm.2008.07.041

[54] Li J, Shao Y, Ding Z, Bao H, Liu Z, et al (2010) Native subunit composition of two insect nicotinic receptor subtypes with differing affinities for the insecticide imidacloprid. Insect Biochem Mol Biol 40:17–22. 20005950 10.1016/j.ibmb.2009.12.003

[55] Lansdell SJ, Millar NS (2000) The influence of nicotinic receptor subunit composition upon agonist, alpha-bungarotoxin and insecticide (imidacloprid) binding affinity. Neuropharmacology 39:671–679. 10728888 10.1016/s0028-3908(99)00170-7

[56] Taillebois E, Beloula A, Quinchard S, Jaubert-Possamai S, Daguin A, et al (2014) Neonicotinoid Binding, Toxicity and Expression of Nicotinic Acetylcholine Receptor Subunits in the Aphid *Acyrthosiphon pisum* . PLoS ONE 9:e96669. 24801634 10.1371/journal.pone.0096669 PMC4011867

[57] Thany S, Lenaers G, Crozatier M, Armengaud C, Gauthier M (2003) Identification and localization of the nicotinic acetylcholine receptor alpha3 mRNA in the brain of the honeybee, *Apis mellifera* . Insect Mol Biol 12:255–262. 12752659 10.1046/j.1365-2583.2003.00409.x

[58] Thany SH, Crozatier M, Raymond-Delpech V, Gauthier M, Lenaers G (2005) Apisalpha2, Apisalpha7-1 and Apisalpha7-2: three new neuronal nicotinic acetylcholine receptor alpha-subunits in the honeybee brain. Gene 344:125–132. 15656979 10.1016/j.gene.2004.09.010

[59] Poitout S, Buès R (1974) Elevage de plusieurs espèces de lépidoptères sur milieu artificiel simplifié. Ann Zool Ecol Anim 2:79–91.

[60] Gemeno C, Haynes KF (1998) Chemical and behavioral evidence for a third pheromone component in a north american population of the black cutworm moth, *Agrotis ipsilon* . J Chem Ecol 24:999–1011.

[61] Gadenne C, Renou M, Sreng L (1993) Hormonal control of sex pheromone responsiveness in the male black cutworm, *Agrotis ipsilon* . Experientia 49:721–724.

[62] Sokal RR, Rohlf FJ (1995) Biometry: The principles and practice of statistics in biological research. New York: Freeman, W.H. 315 p.

